# Diffusion tractography outside the brain: the road less travelled

**DOI:** 10.1007/s00429-025-03062-9

**Published:** 2026-01-05

**Authors:** Kurt G. Schilling, Irvin Teh, Julien Cohen-Adad, Richard Dortch, Ibrahim Ibrahim, Nian Wang, Bruce Damon, Rory L. Cochran, Alexander Leemans

**Affiliations:** 1 Vanderbilt University Institute of Imaging Science, Vanderbilt University Medical Center, Nashville, TN USA; 2 Leeds Institute of Cardiovascular and Metabolic Medicine (LICAMM), University of Leeds, Leeds, UK; 3 NeuroPoly Lab, Institute of Biomedical Engineering, Polytechnique Montreal, Montreal, QC Canada; 4 Functional Neuroimaging Unit, CRIUGM, University of Montreal, Montreal, QC Canada; 5 Centre de recherche du CHU Sainte-Justine, Université de Montréal, Montreal, QC Canada; 6 Mila - Quebec AI Institute, Montreal, QC Canada; 7 Department of Translational Neuroscience, Barrow Neurological Institute, Phoenix, AZ USA; 8 Barrow Neuroimaging Innovation Center, Barrow Neurological Institute, Phoenix, AZ USA; 9 Institute for Clinical and Experimental Medicine, Department of Diagnostic and Interventional Radiology, MR Unit, Prague, Czech Republic; 10 Advanced Imaging Research Center, UT Southwestern Medical Center, Dallas, TX USA; 11 Department of Biomedical Engineering, UT Southwestern Medical Center, Dallas, TX USA; 12 Peter O’Donnell Brain Institute, UT Southwestern Medical Center, Dallas, TX USA; 13 Carle Clinical Imaging Research Program, Stephens Family Clinical Research Institute, Carle Health, Urbana, IL 61801 USA; 14 Departments of Bioengineering and Biomedical and Translational Sciences, University of Illinois at Urbana-Champaign, Urbana, IL USA; 15 Departments of Biomedical Engineering and Radiology and Radiological Sciences, Vanderbilt University, Nashville, TN USA; 16 Department of Radiology, Massachusetts General Hospital, MassGeneral Brigham, Boston, MA USA; 17 Harvard Medical School, Boston, MA USA; 18 PROVIDI Lab, Image Sciences Institute, University Medical Center Utrecht, Utrecht, The Netherlands

**Keywords:** Tractography, Spinal Cord, Heart, Kidney, Brachial Plexus, Prostate, Skeletal Muscle, Peripheral Nerves

## Abstract

Diffusion tractography is a powerful MRI technique for mapping fibrous tissue architecture, traditionally applied to the white matter of the brain. This report surveys the growing application of tractography to anatomical structures outside the brain, a domain that presents both unique challenges and unique opportunities. We examine its use in the heart, spinal cord, peripheral nerves, brachial plexus, kidney, skeletal muscle, and prostate. For each region, we detail the necessary methodological adaptations for acquisition, modeling, and processing, and highlight the unique anatomical information that can be derived for research and clinical applications. While significant challenges remain - spanning technical hurdles like physiological motion and susceptibility artifacts, to biological complexities like lower anisotropy and the interpretation of streamline validity - tractography beyond the brain provides invaluable, non-invasive insights into tissue micro-organization, opening a new frontier for biomedical imaging.

## Introduction

Diffusion tractography is a magnetic resonance imaging (MRI) technique that maps fiber trajectories based on water diffusion (Jeurissen et al. 2019), traditionally used to delineate white-matter pathways *in the brain*. This process reconstructs 3D “streamlines” representing the orientation of tissue structures by leveraging diffusion anisotropy - the tendency of water to diffuse more readily along aligned oriented tissue (i.e., neuronal fibers) than across them. Beyond the brain, many anatomical structures have coherent fiber arrangements (muscle fibers, nerves, ducts, etc.) that can be probed with diffusion tractography. Applying tractography outside the brain presents unique opportunities to visualize microstructural anatomy noninvasively and quantify tissue organization in health and disease. This report surveys foundational and recent research on diffusion tractography in various organs and systems outside the brain, including the heart, spinal cord, peripheral nerves, brachial plexus, kidney, skeletal muscle, and prostate. For each anatomical region, our discussion is structured to first cover why tractography is uniquely valuable for that structure. We then detail the methodological adaptations required for acquisition, modeling, and tractography; describe the information that can be derived and its use in research and clinical applications; and conclude with the current challenges and future directions for the field.

## Heart

### Uniqueness of tractography application

The microstructure of the heart underpins its function as a pump. The myocardium is primarily composed of heart muscle cells known as cardiomyocytes. These are arranged in a complex and intricate 3D architecture that supports the synchronous contractions that propel blood to the rest of the body. In the left ventricular myocardium, a transmural transition in cardiomyocyte orientation is observed, from left-handed helix in the subepicardium (outer layer) to right-handed helix in the subendocardium (inner layer). This transition can be measured using diffusion MRI and visualised in vivo using diffusion tractography (Sosnovik et al. 2009). Changes in the myocardial microstructure are known to occur in pathology, for example, in ischemic cardiomyopathy (Sosnovik et al. 2014) and situs inversus totalis (Khalique et al. 2018). Tractography provides a way to visualize these changes and quantify underlying geometry and microstructure, with the goal to better understand cardiac mechanics, remodeling and disease progression.

### Methods and adaptations

In contrast to the brain, which is relatively static, cardiac motion necessitates specific adaptations, particularly related to the acquisition.

Acquisition: In vivo imaging employs cardiac triggering and potentially respiratory navigators to minimize motion artifacts. This leads to long scan times and fewer slices. This is compounded by shorter T2 in heart relative to brain (van Heeswijk et al. 2012) leading to poor Signal-to-Noise Ratio (SNR). Spin echo methods further require use of motion-compensated diffusion encoding gradients extending echo times, whilst stimulated echo methods require breath-holding. The use of multi-channel array coils can help to improve SNR whilst fast imaging methods such as simultaneous multi-slice imaging can help shorten the acquisition enabling whole heart tractography in vivo (Mekkaoui et al. 2017). Furthermore, the use of high-performance gradients supports acquisition of high-quality in vivo data with shorter TE (Kara et al. 2025; Afzali et al. 2024).

**Fig. 1:**
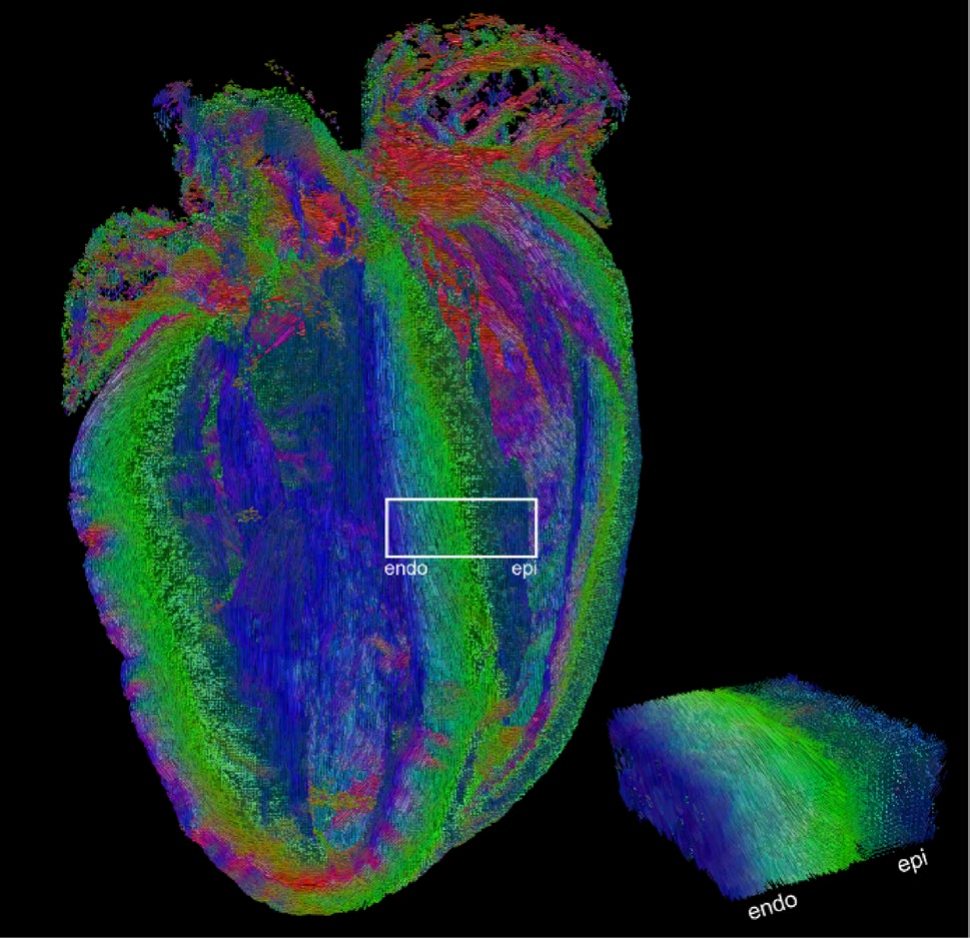
Tractography of ex vivo mouse heart in 4-chamber view acquired at 35 μm isotropic resolution illustrates the transmural transition in orientation of cardiomyocytes in the left ventricular septal wall from subendocardium to subepicardium (highlighted white); underlying data from *(*Teh et al. 2024). Tracks are coloured according to directions: along the heart long-axis (blue), anterior-posterior (green) and septal-lateral directions (red)

Conversely, diffusion tractography of ex vivo hearts in the absence of motion enables the acquisition and reconstruction of data with quality and resolution beyond that achievable in vivo. By optimising methods in Diffusion Tensor Imaging (Basser et al. 1994) (DTI), high quality tractography of ex vivo rat heart was possible at 100 μm isotropic resolution (Teh et al. 2016). In ex vivo mouse heart (Fig. 1), 35 μm isotropic resolution was achievable (Teh et al. 2024), approaching cellular dimensions, and dramatically reducing partial volume and enhancing accuracy of the measurement.

Modeling: For resolving voxels with more than a single dominant cell population, other high angular resolution diffusion imaging (HARDI) schemes e.g. Diffusion spectrum imaging (Wedeen et al. 2005) (DSI) or Q-ball imaging (Tuch 2004) may be considered, along with methods for quantifying microscopic dispersion (Teh et al. 2023) e.g. b-tensor encoding and q-space trajectory imaging (which use complex gradient shapes to probe specific features of tissue structure). DSI has been used to resolve multiple cell populations within a voxel (Sosnovik et al. 2009; Lohezic et al. 2014), however the potential prevalence and significance of such voxels with multiple cell populations remains unclear.

Tractography/Processing: Tractography in ex vivo rabbit hearts in different contraction states has helped elucidate the underlying microstructural changes associated with contraction (Teh et al. 2016). Tractography in these and other species, including pig, sheep and humans, can be used for atlas building and have shown that the transmural variation in cardiomyocyte orientation is well conserved in mammalian species (Lohr et al. 2019; Mekkaoui et al. 2012; Mojica et al. 2020; Lombaert et al. 2012).

### Information derived and applications

​​Tractography provides detailed information on the heart’s microstructural organization. The heart muscle is primarily composed of cardiomyocytes that are connected in a branching syncytium, with secondary aggregations of cells known as sheetlets. Besides Mean Diffusivity (MD) and Fractional Anisotropy (FA), voxel-wise maps of helix angle (HA) and sheetlet angle (E2A) (Stoeck et al. 2016; Ferreira et al. 2014) are often reported. The latter reflect the orientations of the primary and secondary eigenvectors associated with the orientations of cardiomyocytes and sheetlets respectively. Tractography adds potentially important long-range information on the cardiac architecture across the heart.

It is worth mentioning that the use of the term ‘fiber’ is avoided in cardiac diffusion MRI (Dall’Armellina et al. 2024), as cardiomyocytes are discrete and there are no fibers that span multiple voxels at typical clinical resolutions. Furthermore, the use of ‘tracts’ as a descriptor for outputs of tractography can be confused with its anatomical namesake referring to a bundle of nerve fibres. In cardiac tractography, the use of ‘tracks’ or ‘streamlines’ has been recommended instead (Teh et al. 2016).

In research context, supports subject-specific mechanical and electrophysiological modeling as demonstrated in ex vivo rat heart (Fan et al. 2019; Whittaker et al. 2019). These models help investigate the fundamental mechanisms of cardiac function and disease. Tractography also reveals infarct-related remodeling, where scarred regions exhibit reduced FA and disrupted continuity of tracks (Sosnovik et al. 2014).

While still predominantly a research tool, the insights from tractography have clear clinical potential. The ability to characterize infarct-related remodeling could lead to improved non-invasive assessment of scar tissue, while understanding the specific architectural changes in diseased hearts may eventually aid in diagnosis, prognosis, and treatment planning for a variety of cardiac conditions.

### Challenges and future directions

Application of tractography in the heart remains relatively limited, and is constrained by technical limitations. Besides motion, low SNR and long scan times, cardiac DTI often suffers from susceptibility artefacts, respiratory-induced through-plane motion, thick imaging slices, non-isotropic resolution and limited slice coverage. Future advancements are on the horizon for addressing these issues, including faster imaging, distortion correction, improved gradient hardware performance, and machine learning reconstruction. These promise to enhance the prospect of robust and reliable tractography in the heart, opening the way for exciting clinical applications, such as tractography-guided arrhythmia ablation or assessment of myocardial remodeling. 

## Spinal cord

### Uniqueness of tractography application

**Fig. 2:**
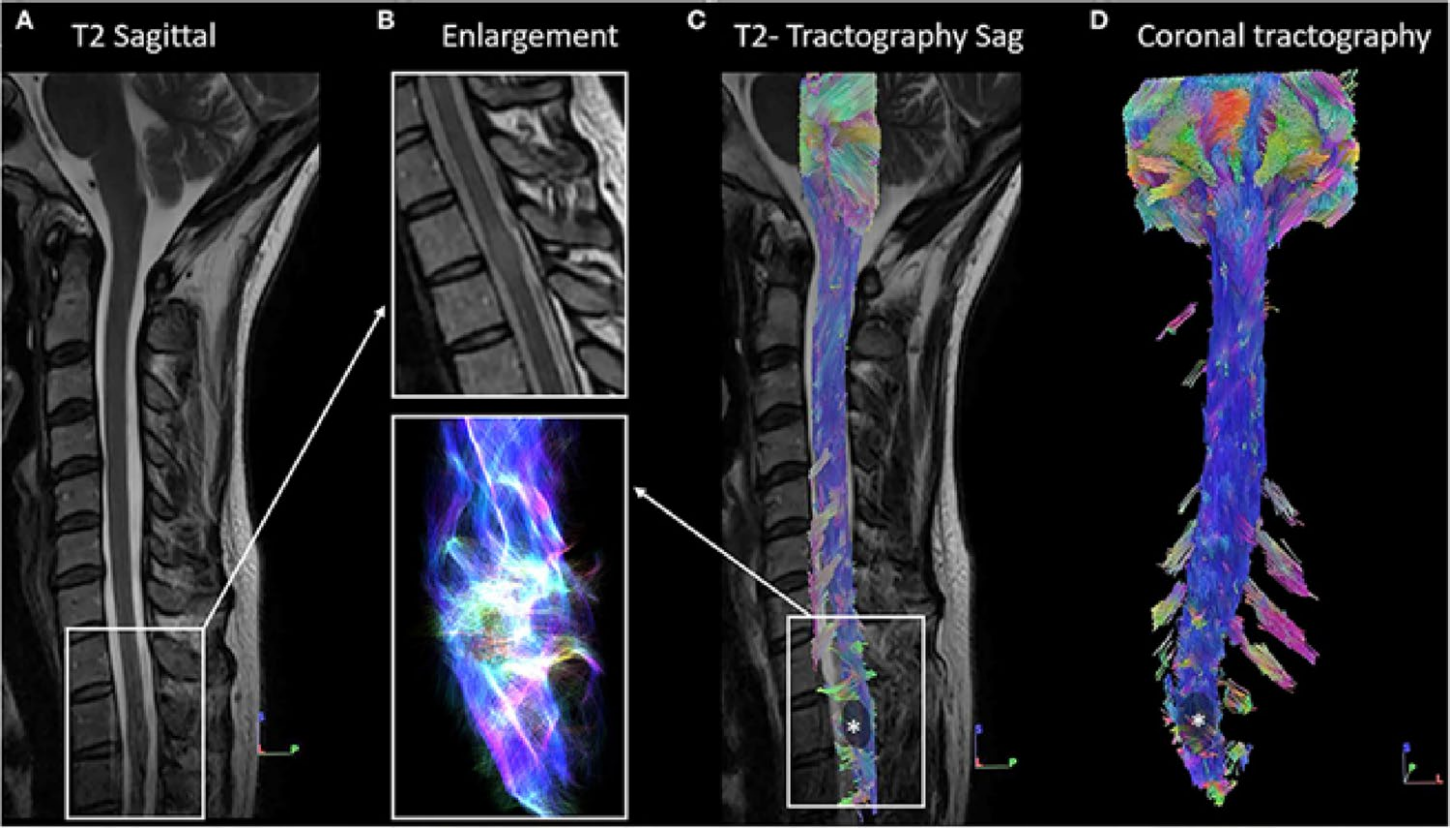
Spinal cord tractography in a patient with intramedullary tumor. Figure and caption reproduced from (Dauleac et al. 2022) highlighting visual deformation and displacement of spinal cord white matter. (A) Sagittal T2-weighted image showing intramedullary isosignal at the Th2-Th3 spine level, associated with an increase in the volume of the spinal cord. (B) 1-Enlargement of panel (A) at the level of intramedullary lesion, on the T2-weighted image. 2-Enlargement of panel (C) on fiber tracking, according to a “line” style rendering performed in DSI Studio, at the level of the lesion. It shows warped fibers within the tumor, without safe access for spinal cord surgery. (C) Overlay of the spinal cord tractography on the sagittal T2-weighted image confirmed fiber deformation at the Th2-Th3 level. (D) Ventral view of spinal cord tractography showing warped fibers at the level of the tumor (indicated by *)

The spinal cord contains white-matter tracts essential for motor and sensory functions. Most of these tracts are coherently aligned along the spinal cord axis. Conventional MRI often fails to reveal the extent of white-matter anomaly, pathology, or injury, but diffusion tractography may enable and improve visualization of tract trajectory and continuity (Van Hecke et al. 2009). This is particularly valuable in spinal cord injury (SCI), where tractography offers a 3D depiction of disrupted or preserved tracts, enhancing diagnosis, prognosis, and monitoring (Nanda et al. 2022). Beyond trauma, it can visualize displacement or infiltration by tumors, aiding surgical planning (Dauleac et al. 2022) (Fig. 2). In short, tractography (and diffusion MRI) provides microstructural and organizational insights not accessible with standard imaging.

### Methods and adaptations

Acquisition: Spinal cord diffusion presents several technical challenges. A typical spinal cord diffusion MRI scan is done with thick axial slices oriented orthogonal to the cord (Cohen-Adad et al. 2021). The in-plane resolution is high enough (about 1 mm) to be able to disentangle the various white matter tracts, while the slice thickness is larger (3–5 mm) to obtain sufficient SNR. However, unlike the brain which is encased in a relatively homogenous skull, the spinal cord’s proximity to bone and other tissues introduces susceptibility artifacts, which can create large distortions on echo-planar imaging (EPI)-based diffusion scans (Stroman et al. 2014). These can be addressed using advanced shimming and reduced field-of-view (FOV) techniques with spatially selective excitation and/or refocusing (Cohen-Adad et al. 2021). Cardiac gating can be used to reduce artifacts related to pulsatile motion of cerebrospinal fluid (CSF). While high resolution acquisitions with anisotropic voxels (voxel sizes ~ 1 × 1 × 5 mm^3^) are common in the research setting, full-cord tractography using isotropic spatial resolution (over a much larger FOV) has also been demonstrated with minimal manual ROI placement (Dauleac et al. 2022).

Modeling: specific adaptations for diffusion modeling in the spinal cord are less common than for acquisition and processing, as standard tensor-based or multi-fiber models are typically applied once the data quality is sufficient.

Tractography/Processing: Motion correction techniques have been adopted specifically for the cord, with slice-wise registration, regularized across slices, outlier detection, and grouping of noisy diffusion volumes to increase SNR during alignment (Xu et al. 2013). Tractography algorithms can then be used to reconstruct 3D fiber paths, enabling the tracing of specific funiculi. These reconstructions, overlaid on anatomical images, enable visual assessment of fiber continuity or deviation. To improve accuracy, exclusion regions (in the form of a cylinder around the cord) have been used to remove false positive connections.

Importantly, because the spinal cord is somatotopically and topographically organized, pathways are distinct in space, and can be derived from existing atlases (De Leener et al. 2018) rather than using tractography directly (McLachlin et al. 2021). Some of the specific pre-processing steps described above can be done using dedicated analysis software such as the Spinal Cord Toolbox (De Leener et al. 2017).

### Information derived

Spinal cord tractography can be used to extract macrostructural and microstructural metrics. The macrostructural metrics include the location and length of specific white matter bundles, and how they connect across the central nervous system. At a microstructural level, tractography can be used to define tract-specific ROIs, within which diffusion-based metrics, such as DTI or multi-compartment models, can be quantified (Witt et al. 2025). For example, at injury sites or lesions locations, FA may be reduced due to disrupted fiber coherence, while MD may vary with edema or scarring.

In a research setting, tractography can be used as a tool for investigating the cord’s structure. It has been applied not only to the cord itself but also to the cervical nerve roots to quantify microstructural integrity in neurodegenerative diseases (e.g., spinal muscular atrophy) (Stam et al. 2019). While tractography can be used to derive ROIs in specific white matter tracts with high sensitivity and reproducibility (Van Hecke et al. 2008), there are limitations. The presence of distortions and the limited spatial resolution of typical diffusion MRI scans could limit the accuracy of tract delineation (Stroman et al. 2014). As an alternative, one can rely on existing MRI templates and atlases of the spinal cord to define white matter tracts (De Leener et al. 2018).

Tractography can also evaluate cord pathology. For example, it can depict fiber continuity or interruptions; in partial injuries, thinning or “feathering” of fibers is observed (Nanda et al. 2022). In the context of oncology, tumors may displace, infiltrate, or truncate tracts, revealing their nature and aiding in treatment planning. Further, metrics like tract count or the fraction of crossing fibers have been used to quantify injury severity and correlate with clinical outcomes (Alizadeh et al. 2018; Park et al. 2022), highlighting potential for prognosis and monitoring.

### Challenges and future directions

Routine clinical use of spinal tractography remains limited by image quality, protocol variability, and lack of standardization. Generic quantitative MRI spinal cord protocols have been developed, and validated, enabling consistent adoption across vendors and sites (Cohen-Adad et al. 2021). Moreover, application or value of tractography over-and-above what can be derived from a white matter atlas has not been convincingly demonstrated. Technical improvements - such as higher field strength (e.g., 7 T), faster acquisition sequences, and advanced diffusion models like multi-tensor or constrained spherical deconvolution (CSD) (Tournier et al. 2007) may enable precise measures of connection/disconnection of specific ascending/descending pathways, distribution of collateral fibers, or nerve roots and nerve integrity.

## Peripheral nerves

### Uniqueness of tractography application

Diffusion tractography has recently been applied to peripheral nerves, including cranial *(*Shapey et al. 2019) and limb nerves (Khalil et al. 2010), offering a noninvasive method to visualize nerve pathways and assess microstructural integrity. Peripheral nerves, composed of myelinated axon bundles, exhibit anisotropic water diffusion similar to CNS white matter. Unlike conventional MRI, which detects gross structural abnormalities, diffusion imaging provides quantitative insights into nerve organization, offering the possibility to detect injuries or neuropathies without overt structural changes. Tractography can delineate nerve continuity, identify disruption due to trauma or inflammation, and support surgical planning by mapping the spatial relationship between nerves and adjacent masses (e.g., distinguishing a nerve sheath tumor from adjacent nerves) (Cauley and Filippi 2013).

### Methods and adaptations

In contrast to the brain, a large and static organ, peripheral nerves are small, mobile structures located throughout the body, often adjacent to tissues like fat and muscle that create significant imaging artifacts.

Acquisition: Imaging peripheral nerves presents numerous technical challenges. The small caliber of nerves, often just a few millimeters to centimeters in diameter, necessitates high in-plane spatial resolution. In clinical scenarios, these demands must be balanced against the need for short scan times, often resulting in the use of thicker slices (i.e., anisotropic voxels). These challenges are further exacerbated by the shorter T₂ values, and associated reduced SNR, of peripheral nerves. Peripheral nerve DTI thus employs high-resolution, fat-suppressed MRI sequences, such as STIR, which are essential to isolate the nerve signal. Imaging typically uses 20–30 diffusion directions to achieve robust tractography despite relatively low anisotropy (Cauley and Filippi 2013).

Specialized coils (e.g., wrist or neck coils) and tailored patient positioning are often required. While high-field MRI systems offer improved SNR, B₀ and B₁ inhomogeneities can negatively affect image quality (which remain a challenge even at clinical field strengths). For example, positioning the upper extremity in the isocenter is difficult, leading to options of either ensuring adequate correction for inhomogeneities or using uncomfortable patient positions (e.g., “superman”, a prone position particularly uncomfortable for patients with traumatic nerve injuries or those susceptible to pressure palsies). Similar challenges exist in the thigh, where large B₁ inhomogeneities are also observed. Because both B₀ and B₁ effects scale with field strength, high-field nerve imaging remains challenging. Cranial nerve imaging presents additional challenges due to CSF pulsation and partial volume effects, which may be mitigated through cardiac gating *(*Shapey et al. 2019).

Modeling: Nerves exhibit distinct diffusion properties compared to the brain due to their larger myelinated axons and the presence of unmyelinated fibers. The preprocessing, modeling, and fiber tracking tools that are standard for brain and spinal cord, however, have not been systematically evaluated in the context of nerve imaging. Quantitative metrics - including FA, MD, axial diffusivity (AD), and radial diffusivity (RD) - are extracted and hypothesized to reflect measures of nerve integrity (Kabakci et al. 2007). Studies have shown that these values correlate with histological features and clinical function, with high FA and AD indicating intact, myelinated fibers (Heckel et al. 2015; Kabakci et al. 2007).

Tractography/Processing: Both deterministic and probabilistic algorithms have been used to reconstruct nerve fibers, such as the sciatic or cranial nerves, in 3D (Zolal et al. 2017; Shapey et al. 2019). During the fiber tracking process, nearby muscles (in limb nerves) (Chen et al. 2019) and white matter structures (in cranial nerves) *(*Shapey et al. 2019) introduce complexity due to their intrinsic anisotropy. This often necessitates the use of lower FA thresholds or alternative diffusion metrics, such as (normalized) quantitative anisotropy (NQA; QA), to enhance tracking specificity. To guide the tracking, regions of interest are placed along known anatomical paths, and seeding strategies are typically anatomically guided (e.g., originating from known nerve root exits) to minimize false-positive tracts.

These combined challenges underscore the need for specialized RF coils, patient positioning protocols, acquisition strategies, and analysis pipelines that are specific to the anatomical region and the nerve being imaged. Given the diversity in nerve size, location, surrounding tissue composition, and physiological motion across the body, protocols must be tailored to address the unique technical and biophysical challenges associated with each nerve region.

### Information derived and applications

With optimized acquisition and analysis, it is possible to achieve accurate and clinically impactful fiber tracks. Tractography of peripheral nerves yields both visual and quantitative insights that can serve as viable preoperative tools, postoperative monitoring tools, or as adjuncts in diagnosis.

**Fig. 3:**
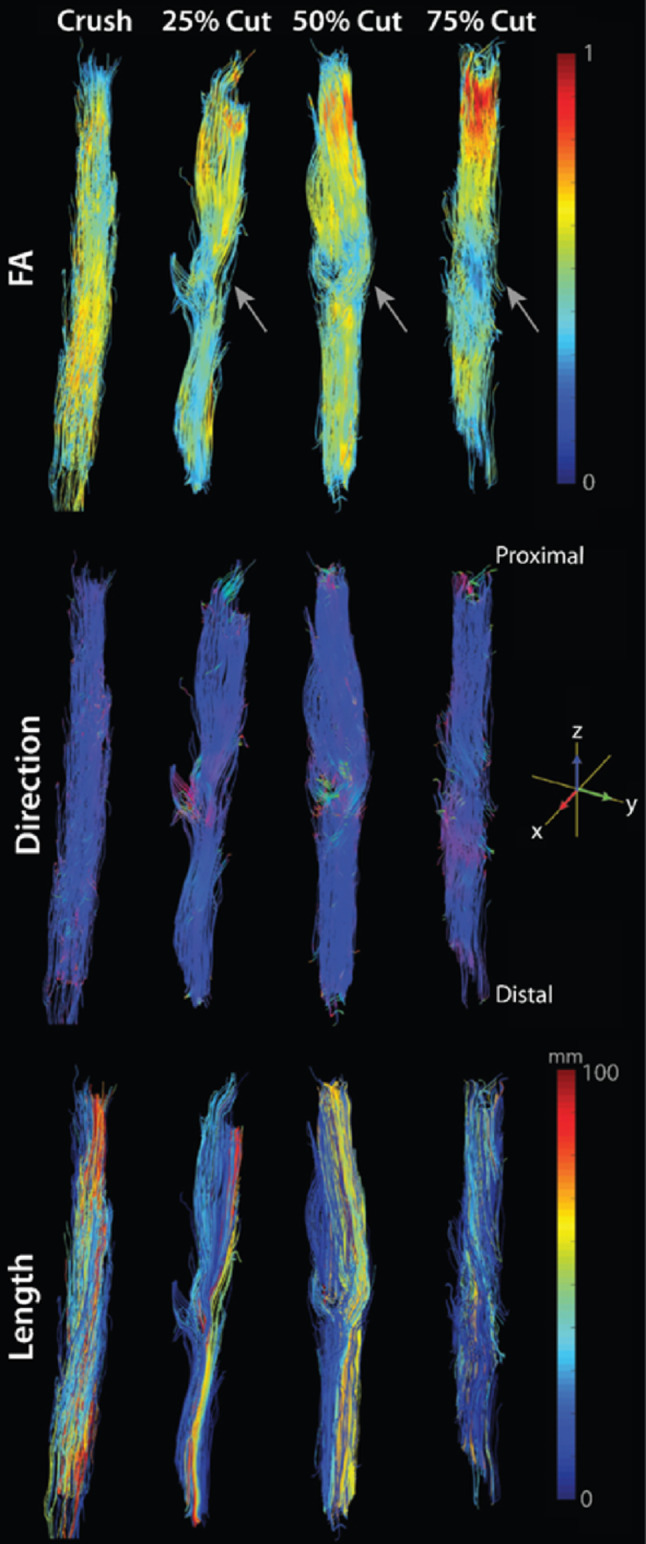
Ex vivo diffusion tractography of rat sciatic nerves four weeks post-injury in crush and partial-cut models (25%, 50%, and 75% transection). Tracts are color-coded by fractional anisotropy (FA; top panels), principal diffusion direction (middle panels), and track length (bottom panels). Partial-cut nerves exhibit focal FA reductions and disrupted directional coherence at lesion sites (gray arrows), whereas crush nerves retain high FA and organized tract orientation. These findings are consistent with substantial recovery in crush injuries by four weeks, compared to ongoing de/regeneration in partial-cut nerves. Figure and caption modified from (Manzanera Esteve et al. 2021)

Tractography of peripheral nerves yields both visual and quantitative insights, largely limited to research applications (Eguchi et al. 2019). For example, tractography confirms nerve continuity and/or reveals abrupt discontinuities following injury (Shapey et al. 2019). Tracts can also show displacement or infiltration due to masses (Taoka et al. 2009). In addition, reduced FA and elevated diffusivity values suggest microstructural damage, such as demyelination or axonal loss (Vos et al. 2015). For example, in multifocal motor neuropathy, nerves show significant thickening (increased cross-sectional area) and lower AD compared to controls, a combination of findings interpreted as both myelin and axonal involvement (Haakma et al. 2017). Following trauma and surgical repair, FA values relate to axon densities (Manzanera Esteve et al. 2019) and report on failed surgeries, successful reoperations, and injury severity (Pridmore et al. 2021). Tract dispersion may also correlate with partial tearing (Manzanera Esteve et al. 2021), and fiber/streamline counts can indicate axonal loss within lesions. These metrics, tracked over time, support assessment of degeneration or regeneration and can be correlated with clinical outcomes or electrophysiological testing, making tractography a viable candidate for clinical translation as an adjunct in both diagnosis and longitudinal monitoring (Fig. 3).

### Challenges and future directions

Challenges include low anisotropy and rapid signal decay in peripheral nerves, which complicate imaging. Muscle tissue adjacent to nerves can mimic nerve diffusion patterns (i.e., anisotropy), risking false-positive tracts. Future advances may include multi-tensor or deconvolution-based models to improve specificity and machine learning tools for nerve segmentation. Standardization of imaging protocols and tractography parameters will be essential for broader clinical adoption. Clinically, more studies are needed to demonstrate that tractography improves decision-making and outcomes.

## Brachial plexus, lumbosacral plexus

### Uniqueness of tractography application

While part of the peripheral nervous system, the brachial plexus (BP) is distinct from individual peripheral nerves. It is a complex network comprising nerve roots (C5–T1), trunks, divisions, and cords that give rise to the upper limb nerves. This intricate anatomy poses challenges for conventional imaging modalities such as MRI and CT, which often struggle to differentiate nerves from surrounding tissues. Advanced MR techniques, including diffusion-weighted imaging (DWI), MR tractography (MRT), and MR neurography (MRN), combined with electromyography (EMG), offer more detailed assessment of both functional and structural characteristics of the BP. Diagnosing brachial plexopathy is particularly demanding and requires interdisciplinary collaboration among neurologists, radiologists, neurosurgeons, and clinical researchers, given its potential long-term impact on motor and sensory function in the upper limb. While EMG, often combined with nerve conduction studies, remains a standard method for evaluating peripheral neuropathy, it can be uncomfortable for patients and provides limited information regarding the precise location and extent of nerve damage. In contrast, MRT techniques such as DTI, DSI, and generalized q-sampling imaging (GQI) enable 3D visualization of nerve fiber architecture by tracking the movement of water molecules in tissue (Vara et al. 2022). MR neurography complements these approaches by providing high-resolution 3D anatomical images of peripheral nerves. These images can be enhanced using Maximum Intensity Projection (MIP) to highlight abnormalities such as entrapment or denervation and to visualize nerve pathways across multiple planes. As a result, these advanced imaging methods are increasingly being incorporated into peripheral nerve evaluation protocols (Khalil et al. 2010). However, in contrast to their established role in brain imaging, MR tractography of the BP and lumbosacral plexus is not yet well established in clinical practice.

### Methods and adaptations

Unlike the brain’s large, well-defined white matter tracts, the brachial plexus is a fine, intricate network of nerves interwoven with muscle and near pulsatile vessels, demanding highly specialized imaging and processing strategies.

Acquisition: MR tractography of the BP is a complex technique that requires careful methodological planning to yield accurate, clinically meaningful results (Vara et al. 2022; Wade et al. 2021). The small size and complex orientation of the BP, coupled with nearby vascular and pulmonary structures, make imaging challenging. Therefore, high-resolution imaging - typically using 3.0 or 7.0 Tesla MRI scanners - is essential.Imaging the BP requires high-resolution diffusion data with multiple b-values and directions of the cervical spine and upper thoracic regions, covering the C5–T1 nerve roots (Vargas et al. 2010). To enhance the SNR, 64-channel head and neck coils are often used in combination with a high-channel phased-array body coil. Multi-shot DWI techniques, such as RESOLVE (Porter and Heidemann 2009), help reduce susceptibility and motion artifacts (Ho et al. 2019). Motion correction strategies may include cardiac gating or the averaging of multiple volumes. The selection of diffusion schemes and parameters - such as the use of multiple b-values (700–1000 s/mm²) and a sufficient number of diffusion directions (e.g., 30, 64, or more) - has a significant impact on the quality of fiber tracking, though these advanced setups are generally more sensitive to motion.

Modeling: While standard DTI-based approaches are common, advanced reconstruction techniques (e.g., GQI, DSI) offer improved resolution and sensitivity to complex fiber orientations found in the plexus (Ibrahim et al. 2022).

**Fig. 4:**
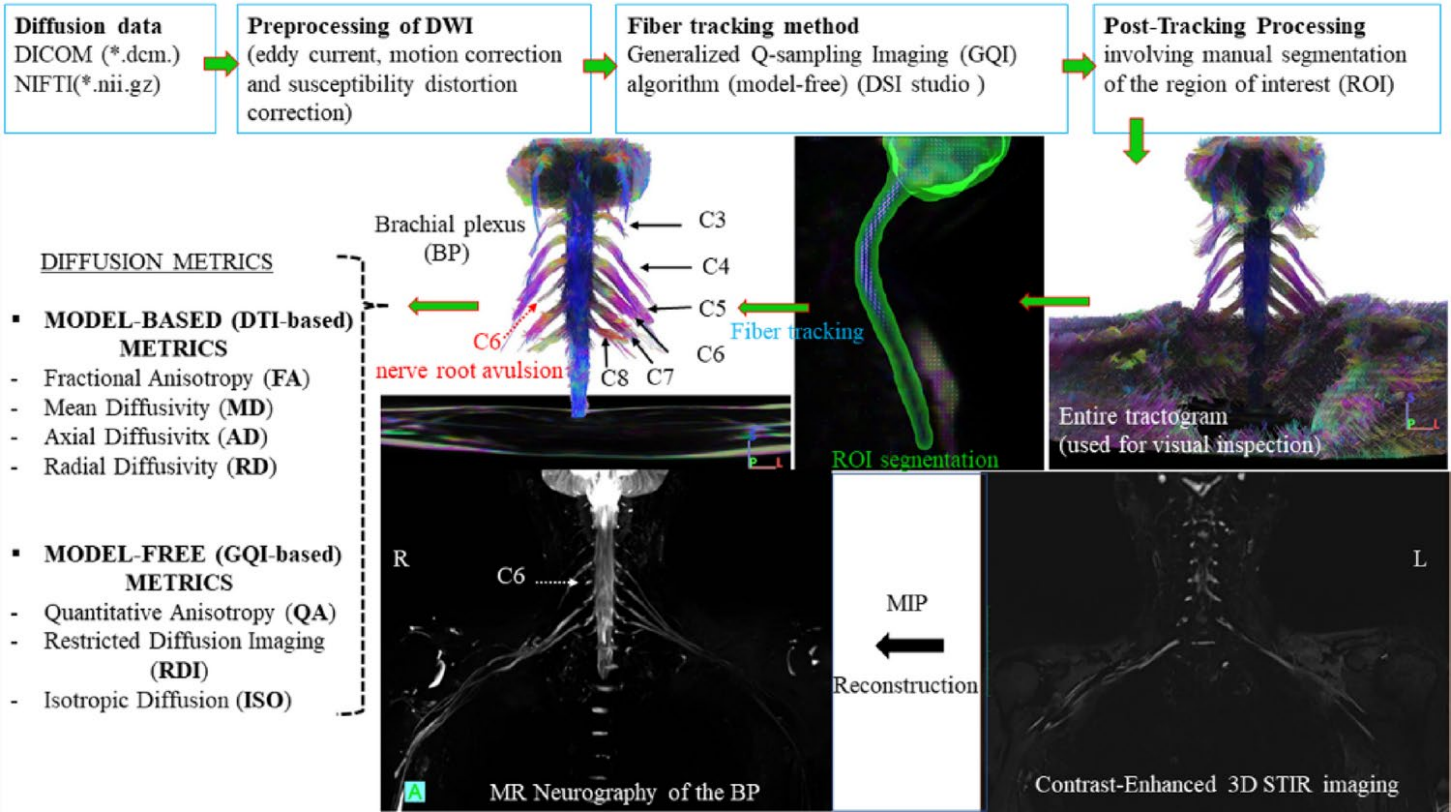
The flowchart illustrates the preprocessing and post-processing of diffusion data in an 18-year-old patient with a right-sided C6 nerve root avulsion. It shows the architectural configuration of the reconstructed fiber bundles (C3–C8, middle row) alongside the corresponding MR neurography (MRN) reconstruction results from a coronal 3D STIR (bottom row). DWI, diffusion weighted imaging; NIFTI, Neuroimaging Informatics Technology Initiative DTI, diffusion tensor imaging, STIR, Short Tau Inversion Recovery. Figure created based on scheme and processing described in (Ibrahim et al. 2024)

Tractography/Processing: Fiber tracking typically begins by selecting a region of interest (ROI) at the spinal cord levels C4–T1, or a broader segment if necessary, which is used as a set of seed points for reconstructing the major root pathways of the BP. Both deterministic and probabilistic tractography algorithms have been employed to visualize these pathways in three dimensions (Wade et al. 2021). To minimize false-positive tractography from adjacent muscle tissue, parameters must be carefully optimized. For example, using a low QA threshold (~ 0.02–0.03) instead of FA, along with angular constraints (30 ~ 50°), helps improve the specificity of nerve tracking and reduce the inclusion of erroneous fibers (Ibrahim et al. 2022, 2024). The resulting tractograms are usually color-coded according to the dominant diffusion direction to help delineate the spatial course of individual nerve roots. Representative MRT and corresponding MRN images of the BP in a patient with a C6 nerve root avulsion are shown in Fig. 4.

The lumbosacral plexus (LSP, L1–S4), which innervates the lower limbs, pelvis, and perineum, can also be visualized (Ibrahim et al. 2021). Compared to the brachial plexus, the LSP exhibits fewer interconnections and fiber exchanges among its constituent neural structures along its course. Its anatomical position - encased by surrounding musculature - provides substantial protection, contributing to a lower incidence of traumatic injury. When injuries do occur, they are most frequently associated with high-energy pelvic ring fractures, particularly those involving the sacrum, as well as with hip dislocations, complex pelvic fractures, or space-occupying lesions within the pelvic cavity (Russell 2006). Tractography of the sacral plexus has been shown to be feasible on 3 T systems, enabling the 3D reconstruction of individual nerve roots from L4 to S3 by using deterministic tracking constrained by anatomical regions of interest (van der Jagt et al. 2012).

### Information derived and applications

Tractography reveals both normal and pathological anatomy of the BP (Alberti et al. 2022). In cases of injury, features such as tract disruption, thinning, or asymmetry help localize the affected roots (Gasparotti et al. 2013), for example the absence of streamlines or fibers may indicate root avulsion (Wade et al. 2020). In tumor-related pathology, tract displacement or truncation can delineate the extent of neural involvement. Quantitative metrics – such as reduced FA or increased diffusivity - highlight microstructural abnormalities, while fiber counts or tract density are often used as indirect indicators of axonal loss or impaired connectivity. This information supports diagnosis, surgical planning, and potentially tracking post-operative recovery.

Similarly, for the lumbosacral plexus, tractography in healthy volunteers provides baseline microstructural values (DTI-derived indices) and can visualize the 3D architectural configuration, including branching toward the pudendal and sciatic nerves (van der Jagt et al. 2012). In a clinical context, such as children with spina bifida, tractography has revealed significant asymmetry, disorganization, and lower diffusivity values in the sacral nerves compared to healthy controls, particularly at the level of the neural tube defect (Haakma et al. 2014). It should be noted that MR tractography of peripheral nerves is still experimental, although research is ongoing.

### Challenges and future directions

MR tractography of the BP and LSP faces several technical and anatomical challenges. These include complex fiber architecture with frequent crossing, and branching, motion artifacts, and difficulty distinguishing nerve fibers from adjacent muscles and vascular structures. The complexity of the BP, combined with its proximity to large blood vessels and the lungs, as well as the small caliber of its nerves, increases the technical difficulty of MR tractography. Similarly, visualization of the lumbosacral plexus is challenging because of its deep pelvic location, small caliber, complex branching, and susceptibility to motion and field inhomogeneities. Future directions involve refining tractography algorithms to better resolve intersecting and diverging fibers, potentially through advanced diffusion models like GQI, CSD or utilizing multi-shell imaging. Validation of MRT findings with intraoperative nerve mapping will be essential to establish clinical reliability. Artificial intelligence (AI)-driven motion correction techniques, as well as faster acquisition protocols, such as simultaneous multi-slice imaging may improve image quality and patient compliance, making the method more feasible in clinical settings.

Further research should explore quantitative indices - such as anisotropy thresholds specific to the BP and LSP, and connectivity metrics - to enhance diagnostic value and reproducibility. Ultimately, large-scale, multicenter studies are needed to confirm tractography’s clinical impact in BP and LSP evaluation, and to define its role alongside standard imaging modalities.

## Kidney

### Uniqueness of tractography application

Unlike the brain, the kidney lacks long fiber tracts characteristic of the nervous system; instead, its microstructure, particularly in the medulla, features aligned tubular structures such as collecting ducts and loops of Henle. These radially oriented tubules generate subtle diffusion anisotropy, which tractography can detect and visualize (Gaudiano et al. 2013). In conditions like chronic kidney disease or fibrosis, this structural coherence may be disrupted. Diffusion tractography offers a noninvasive means of probing these changes, potentially serving as a surrogate for histology by identifying breakdowns in tubular integrity. By mapping diffusion pathways in 3D, tractography provides insight into renal architecture and microstructural health beyond what conventional MRI offers.

### Methods and adaptations

Compared to brain imaging, renal DTI requires adaptations due to abdominal motion and lower anisotropy.

Acquisition: Renal diffusion imaging typically uses respiratory-gated or breath-held acquisitions to account for motion (Wang et al. 2017). Respiratory triggering, single-shot EPI, and coronal imaging planes are commonly used, often paired with fat suppression techniques to reduce signal contamination. Further, lower b-values (400–800 s/mm²) are required to accommodate the kidney’s short T2, perfusion effects, and larger diffusivities (Borrelli et al. 2019).

**Fig. 5:**
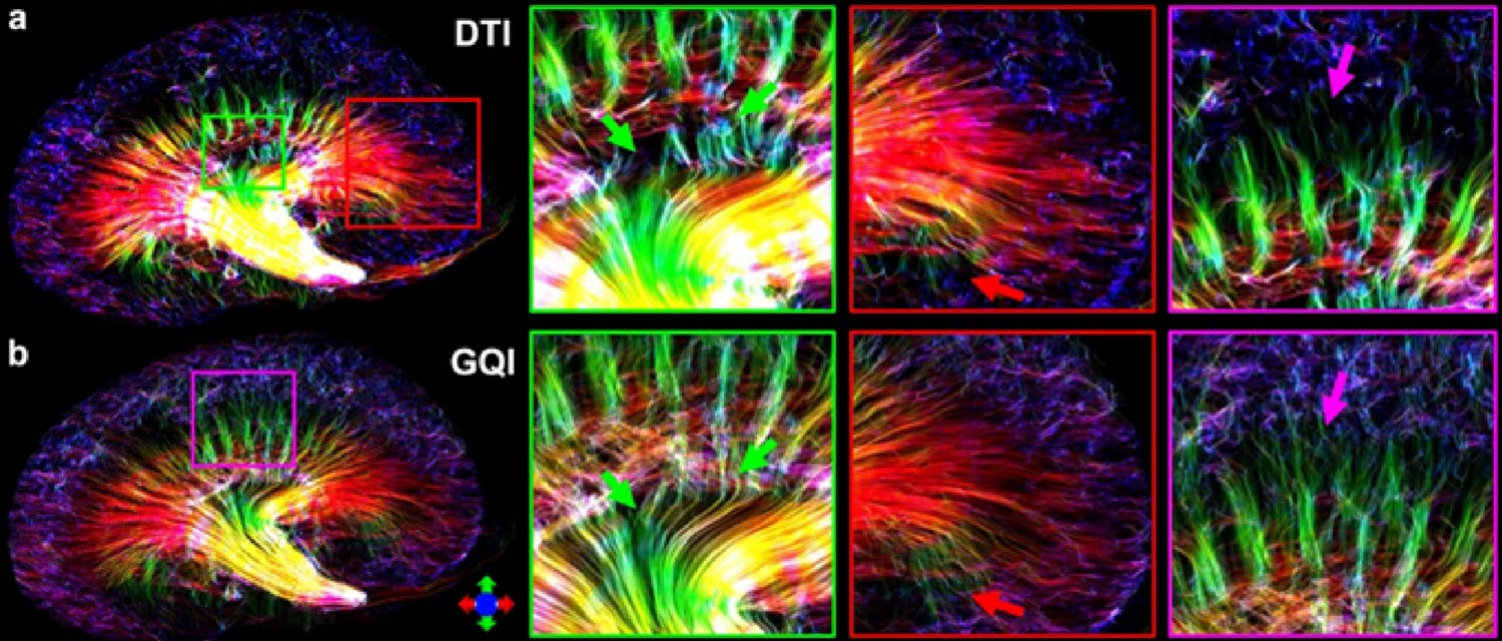
The track density imaging (TDI) derived from diffusion tensor imaging (DTI) and Q-sampling imaging (GQI) models with 46 diffusion encoding directions. Both DTI and GQI were able to track tubules throughout the mouse kidney, while GQI provides more intact tracts in certain regions due to the capability to resolve the crossing fibers (green, red, and purple arrows). (Reprinted from reference (Maharjan et al. 2024)

Modeling: Most diffusion applications in the kidney utilize the DTI model measured across the cortex and medulla. However, to explore the complicated tubular orientation distributions through the whole kidney, alternative HARDI reconstruction techniques (e.g., GQI) have demonstrated superior performance in generating more continuous tracts than the basic DTI model (Fig. 5). In some cases, susceptibility tensor imaging (STI) - a technique that measures how the tissue’s magnetic susceptibility changes as a function of tissue orientation (Li et al. 2017) - has been employed to better capture tubular organization by exploiting susceptibility anisotropy, extending visualization into regions where diffusion MRI proves insufficient. However, a limitation is that STI requires the acquisition of phase images at different subject orientations with respect to the main magnetic field, which may limit its clinical application (Xie et al. 2015).

Tractography/Processing: Tractography typically focuses on the medulla, where anisotropy is highest. Streamlines seeded in the medulla often follow radial paths toward the renal papilla, reflecting the orientation of aligned tubules. In healthy kidneys, this results in regular radial tractograms; in diseased kidneys, the tracts appear sparse or disorganized. Given the medulla’s low FA (~ 0.1–0.2), tractography requires lower thresholds, higher angular resolution, and higher signal averaging to ensure reliable tracking. These tractograms supplement conventional diffusion metrics such as FA or diffusivities, enhancing the structural interpretation of renal health. Tractography has also been applied in transplanted kidneys to evaluate fibrosis or structural disruption.

### Information derived and applications

Tractography in combination with quantitative DTI metrics provides both visual and quantitative assessments of renal microstructure. Visually, tractography in healthy kidneys shows dense, regular radial fibers, while diseased kidneys exhibit patchy, disrupted patterns. Quantitatively, FA is higher in the medulla than the cortex, reflecting aligned tubules versus isotropic tissue, and decreases in FA often signal disease-related disorganization. The directionality of diffusion also provides insight: in healthy states, tracts align from the cortex to the papilla, so any deviations may indicate microstructural distortion.

In a research setting, quantitative measures such as tract density or fiber counts have been described and offer potential biomarkers of fibrosis severity (Xu et al. 2021; Serai et al. 2019; Gaudiano et al. 2013). Clinically, these findings may help characterize kidney integrity and could supplement clinical assessment of renal pathology (Jiang et al. 2021; Serai et al. 2019).

### Challenges and future directions

Renal tractography remains technically demanding due to low anisotropy and motion sensitivity. Advanced acquisition strategies (e.g., free-breathing scans with retrospective motion correction), higher angular resolution, and higher field strengths may improve reproducibility. Interpretation challenges persist, as both fibrosis and inflammation can alter diffusion. Emerging diffusion models (van Baalen et al. 2017) and multi-modal imaging (e.g., STI, elastography) may help differentiate pathologies. Future research should aim to validate tractography against biopsy to establish clinical utility. Ultimately, renal tractography could become a noninvasive biomarker for early detection of disease, staging fibrosis, or monitoring transplant health, offering a new tool for functional imaging of renal microstructure.

## Skeletal muscle

### Uniqueness of tractography application

Skeletal muscles are hierarchically organized, with muscle cells (fibers) being organized into local structural groups called fascicles and distributed functional groups called motor units. Muscle architecture describes the orientation and arrangement of muscle fibers with respect to the muscle’s mechanical line of action and is well matched to the muscle’s mechanical demands. Although understanding muscle architecture is key to biomechanics and physiology, traditional approaches to quantifying muscle architecture used post-mortem dissection or ultrasound, which provide limited (and often, 2D) views (Khalil et al. 2010). Diffusion tensor tractography enables noninvasive, 3D visualization of muscle fiber architecture in vivo (Damon et al. 2002). Water diffuses preferentially along muscle fibers (with the diffusion coefficient being ~ 40% larger along the fiber axis than across it (Cleveland et al. 1976), making DTI well-suited for mapping their orientation (for reviews, see (Heemskerk and Damon 2007; Oudeman et al. 2016; Damon et al. 2017). This method allows measurement of key biomechanical parameters - fascicle length, pennation angle (orientation with respect to the mechanical line of action), and curvature - while also being sensitive to microstructural changes due to injury, edema, or myopathy (Oudeman et al. 2016). Tractography in skeletal muscle provides a direct window into muscle structure-function relationships and pathology, making it a powerful tool for muscle research and a potential clinical aid in diagnosing muscle disorders.

### Methods and adaptations

In contrast to the brain’s highly anisotropic and static white matter, skeletal muscle exhibits lower anisotropy and is often imaged under varying physiological conditions, requiring specific methodological adaptations.

Acquisition: Muscle tractography requires adjustments for motion sensitivity, lower diffusion anisotropy, and shorter transverse relaxation time (T2). To perform muscle tractography, the targeted muscle group is typically imaged at rest, though techniques have also been applied under varying conditions, such as with the joint in different positions (e.g., plantarflexion vs. dorsiflexion) or before and after exercise to measure architectural and physiological changes (Khalil et al. 2010; Froeling et al. 2015; Okamoto et al. 2008; Elzibak and Noseworthy 2014). To improve data quality (Saupe et al. 2009; Damon 2008; Froeling et al. 2013), acquisitions use passive restraints for bulk motion, moderate echo times (45–55 ms), adequate fat signal suppression (Williams et al. 2013; Hooijmans et al. 2025), and moderate b-values (~ 400–500 s/mm²). While multi-shot diffusion sequences can reduce distortion and surface coils can enhance signal in targeted areas, acquisitions typically use a single-shot spin-echo echo-planar imaging (EPI) sequence combined with parallel imaging to reduce acquisition time and artifacts.

Modeling: Modeling in skeletal muscle typically relies on the standard diffusion tensor model, as the primary goal is often the reconstruction of fascicle architecture rather than resolving complex crossing fiber populations.

**Fig. 6:**
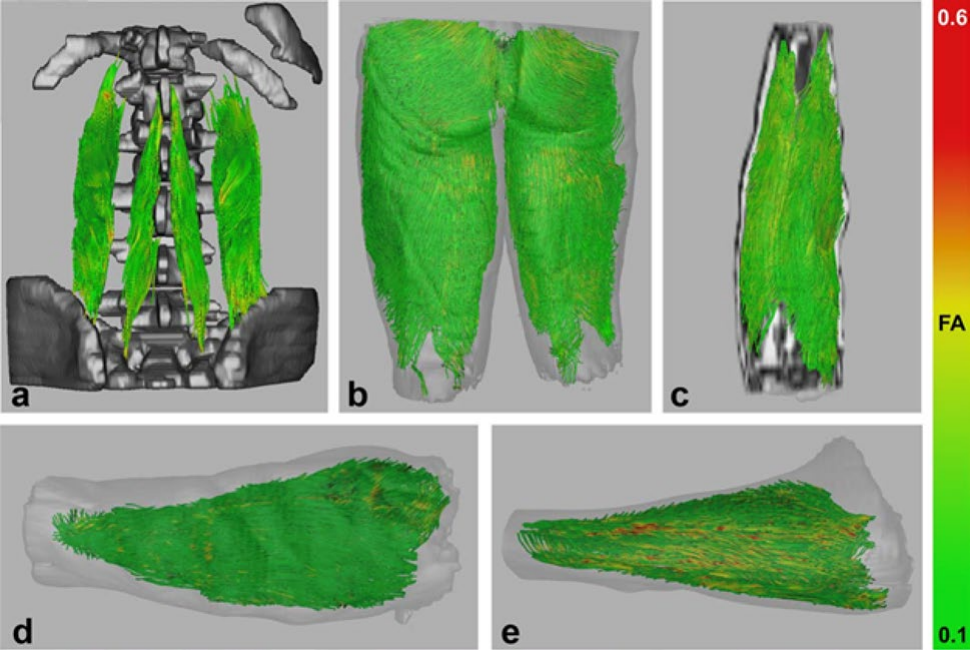
Different examples of skeletal muscle fiber tractography with streamlines color coded based on the fractional anisotropy (FA). a: Fiber tractography of the lumbar erector spinae muscles (Sieben et al. 2016) b: Whole volume tractography of the upper legs. (Froeling et al. 2015) c: Whole volume fiber tractography of the forearm. (Froeling et al. 2012) d, e: Fiber tractography of healthy forearm muscles, next to those of atrophic forearm muscles due to focal/monomelic spinal muscular atrophy, increased FA values can be seen in the affected extensor digitorum muscle, which was also identified by electromyography. (“(PDF) Diffusion Tensor Imaging of Human Skeletal Muscle,” n.d.). Figure reproduced from (Oudeman et al. 2016)

Tractography/Processing: Fiber tracking algorithms are used to reconstruct muscle fascicle trajectories, revealing fascicle-scale architecture patterns. A variety of strategies exist for initiating (seeding) and stopping (terminating) the streamlines. Seeding can be performed at anatomical landmarks or by distributing seed points throughout the entire muscle volume (Damon et al. 2024). Tracts are typically terminated when they reach the muscle boundary or when they encounter anatomically implausible conditions, with common termination criteria including maximum or minimum FA thresholds and a maximum intersegment angle to prevent unrealistic bending (Lockard et al. 2024). From these tractograms, researchers can extract pennation angle by comparing tract direction to the mechanical line of action and measuring fascicle length and curvature directly. And these architecture patterns can be visualized both within and between muscles. For example, in the calf, streamlines seeded in the gastrocnemius visualize fibers curving from tendon insertion toward the knee (Saupe et al. 2009). Diffusion tractography has been implemented in a wide range of muscles Fig. 6 - from the limbs to more complex structures like the tongue and pelvic floor - and in clinical contexts to study muscle injury, denervation, myopathies, and overuse (for a review, see (Oudeman et al. 2016; Khalil et al. 2010). The accuracy of DTI-based architectural reconstructions has been compared to physical dissection and histology in animal and cadaveric tissue (Damon et al. 2002; Schenk et al. 2013).

### Information derived

Tractography provides both anatomical and physiological insights into muscle structure (Damon et al. 2021). Visualization reveals fiber arrangement, including curvature and fiber convergence, such as in bipennate muscles. Pennation angle is one key outcome – tractography enables measurement of the angle at which fibers insert into tendon/aponeurosis in 3D throughout the muscle (Lansdown et al. 2007). Fiber length is another: one can measure the path length of tractography streamlines from one end of the muscle to the other, representing fascicle length (Heemskerk et al. 2009). Curvature, a requirement for mechanical stability (Van Leeuwen and Spoor 1992) and a partial determinant of strain patterns (Blemker et al. 2005), can also be assessed (Damon et al. 2012). In addition, diffusion metrics like FA and MD indicate fiber health (Zaraiskaya et al. 2006; Yanagisawa et al. 2010).

In a research setting, these architectural measures can be used to create biomechanical models. Pennation angle and fiber length can be quantitatively assessed in 3D, informing estimates of muscle function and force generation. This data, combined with muscle volume, can be used to calculate the physiological cross-sectional area (PCSA), a critical determinant of muscle force generation (Lee et al. 2012). For clinical diagnosis and monitoring, diffusion metrics and tract visualization may be valuable. For instance, acute injuries may increase MD and decrease FA due to disorganization, while chronic degeneration reduces both (Esposito et al. 2013; Heemskerk et al. 2007). Tractograms can show fiber disruption, such as terminations at tear sites or abnormal gaps corresponding to scarring, aiding in diagnosis and monitoring of recovery.

### Challenges and future directions

Despite its potential, muscle tractography faces significant challenges. Technical challenges include a low SNR compared to brain imaging, long acquisition times, and a susceptibility to motion and distortion artifacts. Analysis challenges are also prominent; tractography outcomes, particularly fascicle length and curvature, are highly sensitive to user-defined settings like termination criteria and smoothing methods, which complicates the comparison of results between studies (Lockard et al. 2024; Damon et al. 2021). Finally, interpretation challenges include the potential for the diffusion signal to be contaminated by blood volume or blood perfusion, especially after exercise or in inflamed tissue (Oudeman et al. 2016).

Future directions aim to overcome these barriers through protocol standardization and improved and harmonized analysis techniques. The considerable promise of muscle tractography lies in clinical applications such as sports medicine and rehabilitation, where it can noninvasively assess muscle damage and monitor recovery. Further, integrating DTI-derived architectural data into biomechanical models offers a powerful approach to creating patient-specific simulations of muscle function, which could aid in surgical planning and improve our understanding of the relationships among muscle structure and the mechanical and physiological properties of muscle contraction. The latter efforts may be supported by recent applications of registration methods to map muscle deformation due to passive length changes and active shortening (Hooijmans et al. 2025; Guzman et al. 2025).

## Prostate

### Uniqueness of tractography application

The prostate gland is a soft-tissue organ in the true pelvis where diffusion tractography has been studied to map the periprostatic neurovascular fibers that are critical for continence and sexual function (Di Paola et al. 2018), and to characterize the gland’s internal microstructure towards improving the detection of prostate cancer (Hedgire et al. 2016; Gürses et al. 2011; Shenhar et al. 2021). Standard of care prostate MRI consists of at least a biparametric assessment utilizing triplanar T_2_-weighted and high *b*-value (*b* ≥ 1400 s/mm^2^) DWI techniques to detect clinically significant cancer in the transition and peripheral zones, respectively. Dynamic post-contrast enhancement is commonly performed at many centers for a multiparametric examination (Turkbey et al. 2019). Tractography is an attractive adjunctive tool that may be valuable in both aiding prostate cancer detection but also supplement pre-surgical planning. Within the gland, tractography reveals coherent tissue structures, likely corresponding to fibromuscular stroma or glandular ducts. Prostate cancer often disrupts this organization, reducing tract coherence and density. Furthermore, in select patients who have imaging evidence of prostate cancer at MRI, imaging diagnosis and staging involves assessment for the presence of features suggesting extra-prostatic extension (EPE) of the tumor. This is particularly important for patients with organ confined disease who are being considered for curative prostatectomy, as EPE influences the urologist’s surgical approach where they may need to take a wider margin at resection potentially endangering the peri-prostatic neurovascular bundles and informs pre-operative patient counseling. Usually preservation of one or both neurovascular bundles is preferred given their requirement for urinary continence and erectile function. Thus, tractography adds a fiber-based perspective to prostate imaging, complementing existing structural MRI.

### Methods and adaptations

In contrast to the brain, imaging the prostate requires methodological adaptations due to its small size, motion susceptibility and rectal gas.

Acquisition: A pre-examination microenema and administration of an anti-peristaltic agent may help improve image quality by reducing rectal gas and bowel motion, respectively. With advancements in body coils, prostate DTI has been performed without the use of an endorectal coil, at moderate b-values (~ 600–800 s/mm²) and typically up to 32 directions (Di Paola et al. 2018; Shenhar et al. 2021; Hedgire et al. 2016). To improve data consistency, multiple averages and motion correction are often utilized.

Modeling: As the primary goal is often to visualize general tissue structure or major neurovascular bundles, the standard diffusion tensor model is typically sufficient for prostate tractography.

**Fig. 7:**
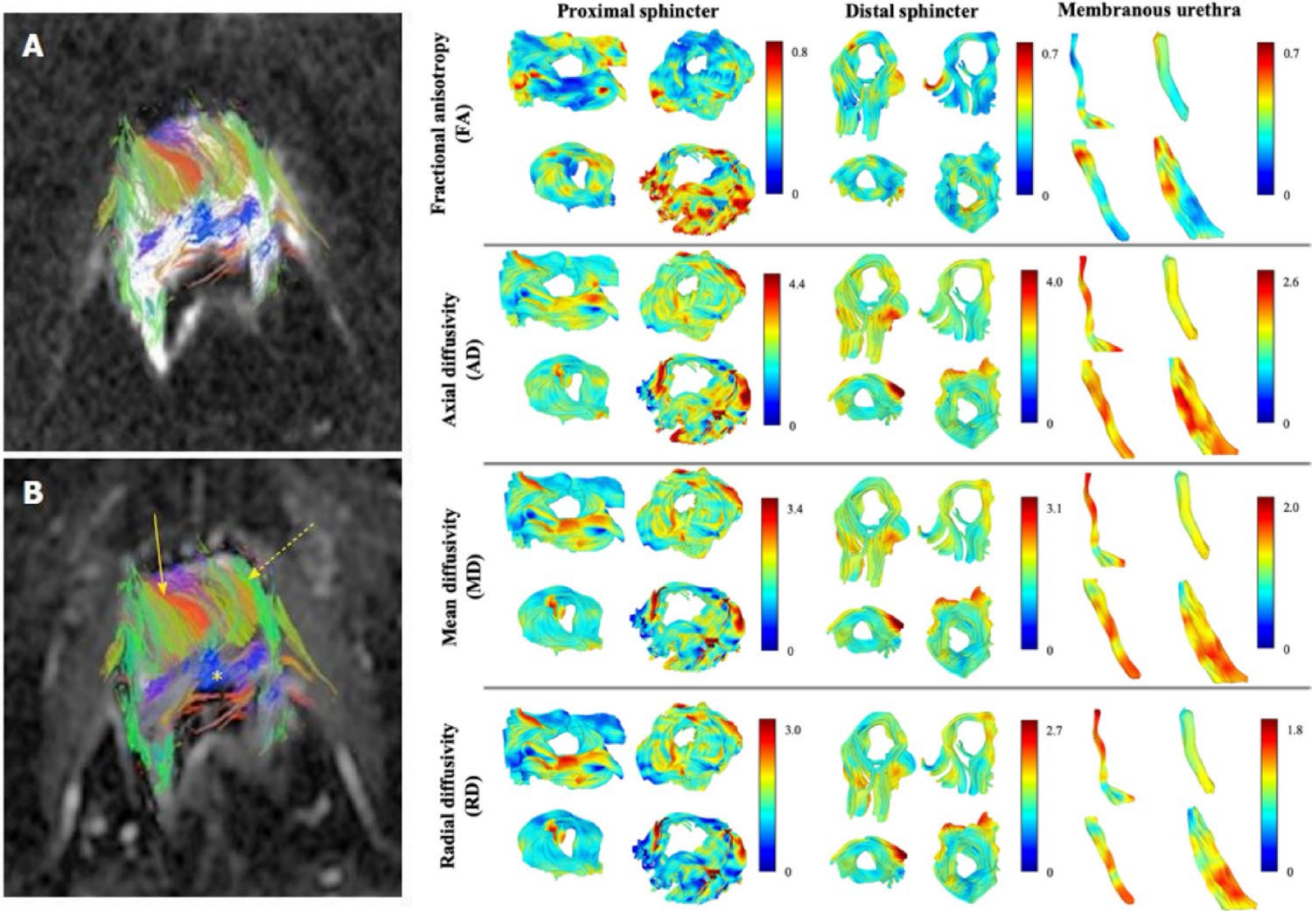
Tractography of the prostate and associated structures. (Left) Figure reproduced from (Hedgire et al. 2016) shows DTI tractography of the prostate is feasible and depicts congregate fibers within the gland. (Right) Figure reproduced from (Verde et al. 2020) shows tractography of structures associated with and nearby the prostate, including patients’ proximal, distal sphincters and membranous urethra, color-coded using the maximum intensity of each DTI metric (from top to bottom, FA, AD, MD, and RD)

Tractography: Corrections for EPI distortion, particularly from rectal gas, are often applied post-acquisition. For neurovascular mapping, seeds can be placed along the posterolateral prostatic capsule to trace nerves coursing from base to apex. Alternatively, seeds can be distributed throughout the prostate to reconstruct tissue structure (Fig. 7). Because prostate FA is low (~ 0.2), tracking thresholds are lowered (0.1–0.15) to capture tissue structure (Hedgire et al. 2016).

### Information derived and applications

While prostate tractography remains largely investigational, it still provides both visual and quantitative data for prostate cancer evaluation. Overall, tractography reveals that the prostate has intrinsic anisotropic structure (Hedgire et al. 2016; Finley et al. 2012), and changes in tract patterns may serve as biomarkers for disease. Tract maps can qualitatively highlight regions of disrupted architecture, and quantitative metrics like tract-density can be measured (Kitajima et al. 2014).

In research settings, tumor regions have shown reduced tract density compared to normal tissue, suggesting microstructural disorganization (Hedgire et al. 2016). Interestingly, some studies noted increased fiber density in aggressive cancers (Gholizadeh et al. 2019), potentially reflecting tumor-induced neurogenesis (Magnon et al. 2013; Olar et al. 2014). For clinical evaluation, tractography of neurovascular bundles can show nerve trajectories along the prostate and reveal their potential displacement by tumors, which can aid in planning for nerve-sparing surgery (Di Paola et al. 2018; Kitajima et al. 2014; Ream et al. 2016).

### Challenges and future directions

Key challenges include low tissue anisotropy, motion artifacts, rectal gas, and short tract lengths. Improved hardware, faster sequences, and motion correction strategies are needed to enhance tract reliability. Standardizing tract-based metrics, and determining which features (diffusion-based features, or features of the streamlines) will be essential for clinical use. Integrating tractography with multiparametric MRI may enhance diagnostic specificity. Deep learning image reconstruction of prostate DT image sets is also expected to be an area of active research in the near term. Additionally, tractography may help monitor post-treatment changes, assessing microstructural recovery or nerve preservation. As imaging technologies evolve, prostate tractography may complement existing modalities by providing spatially resolved, fiber-based insights into cancer and neuroanatomy.

## Conclusion

In the brain, diffusion tractography is a cornerstone technique used to delineate the connections, course, and trajectory of major white matter pathways. From these streamlines, researchers extract microstructural metrics (reflecting axonal organization, density, etc.) and macrostructural features (e.g., tract length, cross-sectional area, volume) to infer connectivity strength, coherence, and anatomical organization. Extending this beyond the brain opens a new frontier, where tractography reveals the microarchitecture of tissues not traditionally studied using these techniques.

This review demonstrates that tractography, when adapted outside the brain, offers novel capabilities across diverse anatomical systems. In the heart, it reveals the transmural helical arrangement of myocardial fibers and quantifies remodeling in disease. In the spinal cord, it visualizes longitudinal white-matter continuity and injury-related disruptions. Peripheral nerve and brachial plexus tractography map fine nerve pathways in 3D, aiding in diagnosis and surgical navigation. Muscle tractography quantifies fascicle orientation and pennation, key to understanding biomechanical function and injury. In the prostate, tractography provides a unique window into periprostatic nerve anatomy and intraglandular structural coherence, potentially improving tumor characterization and surgical precision. Even in the kidney, where organized tubules yield only subtle anisotropy, tractography has revealed medullary organization and its breakdown in disease.

Across these domains, tractography provides two critical advantages: (1) non-invasive, in vivo visualizations that reflect tissue architecture and spatial relationships, and (2) quantifiable metrics sensitive to structural integrity and pathological change. The extracted features - tract coherence, density, length, curvature, and direction - serve as potential biomarkers of health, injury, or disease progression.

However, the transition from brain to body introduces new challenges. Organs often exhibit lower diffusion anisotropy, are more prone to physiological motion, and require high-resolution imaging to capture fine structures. As such, adaptations in acquisition (e.g., respiratory gating, specialized coils), modeling (e.g., multi-compartment diffusion, low-anisotropy tracking thresholds), and processing (e.g., tissue-specific seeding, motion correction) are essential. Moreover, standardization and validation are crucial: tractography-derived features must be shown to correspond to real anatomical and functional endpoints through correlation with histology, intraoperative findings, or clinical outcomes.

Looking ahead, the integration of tractography into routine clinical workflows depends on several advances. Technical improvements in MRI hardware (e.g., stronger gradients, high-channel coils), reconstruction (e.g., deep learning and compressed sensing), and motion robustness will be pivotal. The development of normative atlases and organ-specific tractography protocols will facilitate broader application. Furthermore, combining tractography with complementary modalities (e.g., multiparametric MRI, electrophysiology, computational modeling) can enhance diagnostic power and mechanistic insight. Finally, it is important to understand what features of these streamlines are relevant, and what biological or anatomical features they reflect.

In summary, diffusion tractography beyond the brain has demonstrated its potential to noninvasively map structural organization across a spectrum of tissues. It yields biologically meaningful metrics and clinically relevant visualizations, and it opens new paths for understanding tissue function, injury, and disease. The voyage of diffusion tractography, from the brain to the rest of the body, has only just begun, and each organ presents new opportunities for discovery, innovation, and ultimately, translational impact.

## Data Availability

No datasets were generated or analysed during the current study.
